# Implementation of US Post-Acute Care Payment Reform and COVID-19 Policies: Examining Experiences of Health System Leaders, Staff, Patients, and Family Caregivers—A Protocol

**DOI:** 10.3390/ijerph20206959

**Published:** 2023-10-23

**Authors:** Natalie E. Leland, Stephanie A. Rouch, Rachel A. Prusynski, Amanda D. Shore, Hannah Kaufman, Lorelei P. Hoover, Tracy M. Mroz, Janet K. Freburger, Debra Saliba

**Affiliations:** 1 Department of Occupational Therapy, School of Health and Rehabilitation Science, University of Pittsburgh, Pittsburgh, PA 15219, USA; rouchsa4@upmc.edu (S.A.R.); ams728@pitt.edu (A.D.S.);; 2 The Wolff Center, University of Pittsburgh Medical Center, Pittsburgh, PA 15213, USA; 3 Department of Rehabilitation Medicine, University of Washington, Seattle, WA 98195, USA; rachelp1@uw.edu (R.A.P.); hkaufman@uw.edu (H.K.); tmroz@uw.edu (T.M.M.); 4 Department of Physical Therapy, School of Health and Rehabilitation Science, University of Pittsburgh, Pittsburgh, PA 15219, USA; janet_freburger@pitt.edu; 5 Veterans Affairs (VA) Greater Los Angeles Geriatrics Research Education and Clinical Center (GRECC), Los Angeles, CA 90095, USA; saliba@rand.org; 6 Borun Center, University of California, Los Angeles, CA 90095, USA

**Keywords:** home healthcare, skilled nursing facility, mixed methods, multiple case study, patient-driven payment model, patient-driven groupings model, post-acute care, COVID-19

## Abstract

In fiscal year 2020, new national Medicare payment models were implemented in the two most common post-acute care settings (i.e., skilled nursing facilities (SNFs) and home health agencies (HHAs)), which were followed by the emergence of COVID-19. Given concerns about the unintended consequence of these events, this study protocol will examine how organizations responded to these policies and whether there were changes in SNF and HHA access, care delivery, and outcomes from the perspectives of leadership, staff, patients, and families. We will conduct a two-phase multiple case study guided by the Institute of Medicine’s Model of Healthcare Systems. Phase I will include three cases for each setting and a maximum of fifty administrators per case. Phase II will include a subset of Phase I organizations, which are grouped into three setting-specific cases. Each Phase II case will include a maximum of four organizations. Semi-structured interviews will explore the perspectives of frontline staff, patients, and family caregivers (Phase II). Thematic analysis will be used to examine the impact of payment policy and COVID-19 on organizational operations, care delivery, and patient outcomes. The results of this study intend to develop evidence addressing concerns about the unintended consequences of the PAC payment policy during the COVID-19 pandemic.

## 1. Introduction

Post-acute care (PAC), a key component of the United States (US) healthcare continuum, is designed to bridge the transition from short-term acute care hospitals back to the community. Annually, over 40% of Medicare beneficiaries are discharged from acute care hospitals to care by a PAC organization to foster continued recovery [1,2]. PAC provider types include skilled nursing facilities (SNFs), inpatient rehabilitation facilities, long-term care hospitals, and home health agencies (HHAs) [1,2]. SNFs and HHAs provide the bulk of PAC services under Medicare Part A, accounting for ~80% of PAC spending in the US [3]. Skilled services available in PAC include nursing, social work, and rehabilitation (i.e., physical therapy, occupational therapy, and speech–language pathology). Outcome metrics for PAC focus on improving function and ameliorating adverse events (e.g., falls, rehospitalization) to facilitate safe community discharge—an outcome prioritized by patients and families, providers, and the Centers for Medicare & Medicaid Services (CMS) [4].

### 1.1. History of PAC Spending

In the 1980s, there were a series of efforts to control escalating hospital spending, which included the implementation of diagnosis-related groups and acute care payment reform, which incentivized shorter hospital stays and a shift of patients from hospitals to lower-cost PAC healthcare settings (e.g., SNFs) [5]. This hospital payment policy accelerated the growing use of PAC and shifted Medicare spending to these services [6]. Given this growth, the Balanced Budget Act of 1997 initiated the implementation of prospective payment for PAC in an effort to control PAC spending by transitioning away from a cost-based model [7]. While the prospective payment systems varied for SNFs and HHAs, the underlying design of both required the classification of patients into different resource utilization groups based on illness severity.

Nonetheless, PAC spending continued to rise after the 1997 policy [8,9,10], doubling from USD 26.6 in 2001 to USD 57.5 billion in 2019, of which Medicare SNF services totaled USD 27.6 billion and HHA services cost USD 18 billion [11]. These rising PAC costs were largely driven by the number of patients being classified in the high-intensity therapy tier in SNFs and HHAs. Under the SNF and HHA prospective payment systems, delivering high-intensity therapy services yielded the largest payments in SNFs and HHAs [12,13,14]. The majority of SNF and HHA patients were in the highest reimbursement categories, which raised concerns about patient selection and inappropriate use of and overpayment for therapy relative to other services [9,15,16,17]. Further, therapy volume was the primary source of geographic variation in PAC costs, suggesting that increased use of therapy services was financially motivated to maximize profits in certain regions [18,19,20,21,22,23].

To address concerns about the financial incentive to deliver high-intensity therapy services, CMS implemented new PAC payment models in SNFs and HHAs [15,16,17,24]. The Patient-Driven Payment Model (PDPM) for SNFs was implemented in October 2019. The Patient-Driven Groupings Model (PDGM) for HHAs began in January 2020. These new payment models were designed to remove volume-based financial incentives and instead base payment on patient characteristics in an effort to foster individualized patient care [9]. PDPM payments to SNFs take into account the admitting diagnosis (i.e., ICD-10 coding), comorbidities, functional status, and cognitive status [25,26]. Similarly, new 30-day episode rates under PDGM for HHAs are based solely on patient characteristics at the start of care, including principal diagnosis, admission source (community or institution), the timing of the 30-day period (i.e., whether it was the first 30-day period or subsequent 30-day period), functional status, and comorbidities. The new payment models in both settings have removed therapy utilization as a determinant of payment, thus removing the financial incentive to deliver therapy. However, without this incentive, concerns have been raised about continued access to appropriate care.

PAC community partners raised concerns that the new payment models would reduce therapy services and perpetuate existing inequities, thus negatively impacting patient outcomes [27,28,29,30]. Further, while acknowledging issues within the prior payment models, technical expert panel members and other community partners questioned whether the new policies would alleviate or exacerbate existing disparities [31,32,33,34,35,36], given the absence of quality as a component of the new payment models. Without incentives to deliver rehabilitation, these services may be reduced to control costs, which could be detrimental to certain vulnerable populations and diagnostic subgroups [25]. These concerns were reinforced by CMS-funded impact analyses conducted prior to the implementation of these programs [25,29,37]. These analyses projected that some patient groups would provide SNFs and HHAs with reductions in reimbursement, including beneficiaries (a) over age 75, (b) those diagnosed with mild to moderate cognitive impairment, and (c) those with an admission diagnosis of joint replacement, hip fracture, dementia/Alzheimer’s, stroke, or neurological conditions (e.g., Parkinson’s disease). Conversely, analyses suggest that payments to SNF and HHA organizations for dual-eligible and rural patients may increase.

### 1.2. Emergence of COVID-19

The emergence of the COVID-19 pandemic and subsequent public health emergency occurred soon after PDPM and PDGM were implemented and significantly affected PAC [38]. The public health emergency declaration first issued on 31 January 2020 initiated changes in healthcare operations and utilization, such as hospitals pausing elective surgeries (e.g., total joint replacements) to free up hospital beds for COVID-19 cases [39,40]. Simultaneously, national, state, and regional agencies mandated elevated safety and infection control policies, such as no-visitation policies in SNFs, a moratorium on group activities (e.g., SNF patient group activities, SNF and HHA staff in-person staff meetings), and use of personal protective equipment (PPE) to control viral spread [41,42,43,44]. Working conditions for administrators and frontline staff during the COVID-19 pandemic resulted in increased stress, burnout, and turnover, which likely also impacted the provision of high-quality PAC [42,44,45,46,47,48].

Driven by the need to prioritize infection control and safety, PAC providers altered care delivery and reallocated resources [43,49,50]. Further, concerns about infection control may have led providers to limit key services (e.g., therapy) in order to reduce the number of providers interacting with patients and to conserve available PPE [42,43,51,52,53]. In addition, waivers implemented by various payors during the public health emergency allowed payment for expanded use of telehealth, which may have accelerated telehealthcare innovations [54]. Yet, little is known about how organizational operations and care delivery have changed [55,56]. In response, this study seeks to respond to these gaps in the PAC literature within the context of the emergence of COVID-19.

### 1.3. Conceptual Model

**Figure 1:**
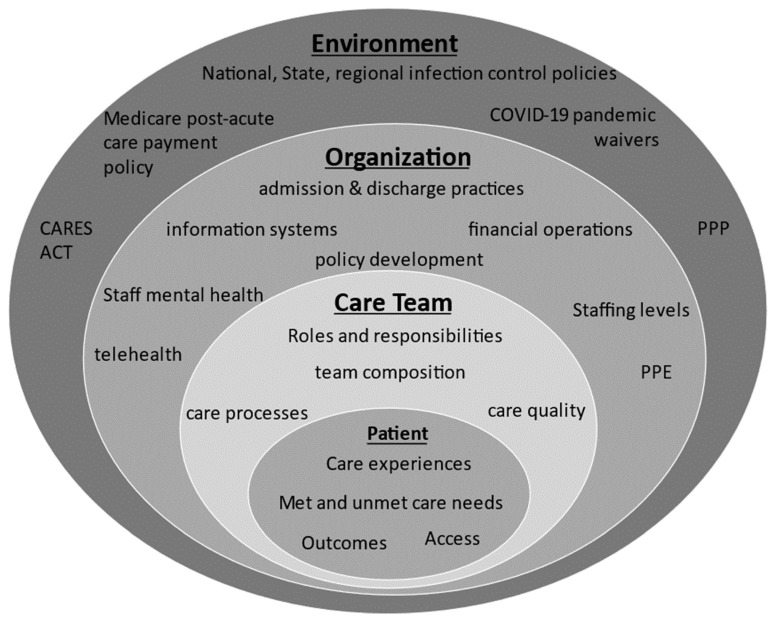
A conceptual model to examine payment reform and COVID-19.

This study protocol is informed by the Institute of Medicine’s model of healthcare systems [57], which is presented in Figure 1. The framework describes a four-level, nested healthcare system that includes the individual patient nested within the care team, within the organization, and the environment in which the organization is situated. The care team consists of healthcare providers, the patient’s family, and the patient. The organization includes the physical infrastructure and resources. This model depicts the environment to include regulations, policies, and economic environment (e.g., local, state, national level) that influence the way the healthcare system functions.

### 1.4. Research Objectives

Given the confluence of new PAC payment models and the COVID-19 pandemic, there is a need to understand the variation in organizational responses of SNFs and HHAs to elucidate unintended consequences (e.g., perpetuation of inequities) and determine factors that contributed to these consequences to guide future research and policy. Thus, the overarching objective of this study protocol is to examine the impact of PAC payment reform and COVID-19 on SNF and HHA operations, staffing, care delivery, and quality from the perspectives of organizational leadership, staff, patients, and family caregivers. This study protocol details our efforts to explore perspectives of organizational processes, barriers, and facilitators to high-quality care, and patients’ and family caregivers’ care experiences after the implementation of payment reform and the evolution of the COVID-19 pandemic.

## 2. Methods and Analysis

This study protocol describes the design of an exploratory multiple case study design. This approach was chosen as it is intended to obtain an in-depth understanding of the phenomenon (i.e., the impact of payment reform and COVID-19) within the organizational context of each SNF and HHA [58,59,60,61]. Unlike traditional descriptive qualitative studies that use data saturation of a homogenous group to guide sample size, multiple case study design aims to maximize variation across organizational contexts [62,63]. This approach allows for a comprehensive view of a heterogeneous sample to identify similarities and differences across organizational contexts to better understand the who, what, why, when, and how of the phenomena [63]. This study includes two phases. Phase I will focus on capturing the perspectives of a national purposive sample of administrators. These insights will provide the opportunity to understand SNF and HHA operations, staffing, care delivery, and care quality from the perspectives of leadership across a wide range of organizations. Phase II will focus on a sub-sample of SNFs and HHAs from Phase I to gain a better understanding of the phenomena from multiple perspectives within each organization. These insights are intended to augment evidence captured from leadership to understand the experiences of frontline staff, as well as patients and family caregivers. Further, given concerns about these payment policies perpetuating healthcare disparities, Phase II sampling will allow us to contribute evidence on PAC (in)equity.

### 2.1. Defining Cases for Phase I (Administration Perspectives)

Given the preponderance of evidence that associates quality with patient outcomes, we will construct three cases to reflect our multiple case study design. The construction of each case will reflect a different level of quality performance and include a maximum of 50 organizations. Phase I cases will be constructed as high-, medium-, and low-quality based on CMS five-star publicly reported quality performance ratings from the SNF Quality Measures Domain and the HHA Patient Quality Rating for the 2019 calendar year [64,65,66,67,68,69,70,71,72,73,74]. Organizations with four- and five-star ratings will be categorized as “high quality” cases. Organizations with a three-star rating will be categorized as “medium quality” cases. Organizations with one- and two-star ratings will be categorized as “low quality” cases.

### 2.2. Phase I Sampling Frame and Eligibility

A maximum of 150 organizations representing each setting will be identified, which includes a maximum of 50 organizations per case. Within each case, we will maximize variation across three domains (Table 1), including (a) regional location (Northeast, Midwest, South, or West), (b) profit status (for profit or non-profit/government), and (c) urban vs. rural location.

To identify eligible organizations within the sampling frame and later describe participating organizations, we will leverage four publicly available organization data sources, including the CMS Provider of Services File [75], Medicare PAC and Hospice Provider Utilization and Payment Public Use Files [76], Medicare Care Compare for SNF [77], Medicare Care Compare and for HHA [78], Payroll-Based Journal [79], and LTCFocus [80]. We will merge these public data sources by provider identifier. The final databases for each set will include over 15,000 SNFs and 11,700 HHAs to categorize organizations into cases (i.e., high, medium, low quality) and then sample frame specifications based on the three domains (i.e., region, profit status, urbanicity).

Within each cell of the sampling frame (e.g., low quality, for-profit urban SNFs in the Northeast region of the US), we will then use additional organizational characteristics as needed to prioritize 15 initial organizations to contact for recruitment. These additional variables include the state where the organization is located, the use of contract or in-house therapy, and the percentage of patients dually eligible for Medicare and Medicaid. Given the variation in when COVID-19 hit different regions of the US, we will also create a county-level measure for COVID-19 using data from USAFacts [81]. To construct this measure, we will (a) determine the county in which the organization is located, (b) identify the 2020 COVID-19 per capita rate for that county, and (c) create an indicator for organizations located in counties in the top quartile of per capita COVID-19 case rates in 2020. The study team will examine the distribution of these variables and modify the order as needed to ensure variation within cells. This application of these additional characteristics will not be applied across all cells of the sampling frame due to the small number of organizations in some cells. Once fifteen SNFs and HHAs within each cell are prioritized with maximum variation, the top five SNFs or HHAs from each cell will be selected from each region for initial recruitment.

To ensure variation in completed interviews according to the sampling frame [82], the study team will prioritize initial interview scheduling with SNFs and HHAs in sampling frame cells with fewer organizations. We will also track, on a weekly basis, the number of SNFs and HHAs, within each cell, that schedule and complete interviews. Additional SNFs and HHAs will be added to the recruitment list each week as prior organizations decline to participate, are a no-show for an interview, are unresponsive to efforts to reschedule, or are unresponsive to initial recruitment efforts. This approach includes targeting no more than ten organizations per setting within one cell of the sampling frame at any given time.

For each organization that agrees to participate, we will seek one member of leadership to participate in the interview that can speak to the organizational response to the PAC payment policy and the evolution of the COVID-19 pandemic. Examples of job roles that can speak to the organization’s response include the administrator, center executive director, director of nursing, home health agency director, home health manager, and director of operations.

### 2.3. Phase I Recruitment Effort

Phase I recruitment of administrators representing organizations prioritized in our sampling frame will involve creating flyers, letters, newsletter summaries, and a study website with community partner input. The study team will collaborate with national industry organizations to (a) obtain feedback on materials and (b) disseminate information to their membership about the study to increase visibility and institutional support. From our various informational materials, each organization will select the format and content that aligns with their membership communication strategies, such as flyers, to attach to weekly email blasts or study summaries to include in member newsletters and member communication boards.

The study team will use public Information to construct contact information for each organization that is prioritized based on the aforementioned sampling frame prioritization process. Team members will use this information to call sites to obtain administrator contact information in order to mail, fax, or email a description of the study and recruitment materials. These materials will include (a) a personalized letter summarizing the objective of the study, (b) contact information for the project coordinator to answer study-related questions, and (c) a recruitment flyer. Within seven to ten days of the initial contact, the study team will follow up with a phone call or email to determine the administrator’s interest in participating.

The study team will complete recruitment calls considering (a) the time zone in which the organization is situated as well as (b) the study teams’ prior knowledge of SNF/HHA workflow when administrators may be more busy than normal and unable to take a phone call (e.g., Fridays when hospitals are often discharging patients to SNFs/HHAs before the weekend). Email follow-up attempts will be completed early in the traditional work week (i.e., Monday and Tuesday) to increase the probability of the administrator attending to the email sent. Following the IRB-approved protocol, the study team will make up to three to five contacts to schedule an interview. If the administrator is unavailable, a voicemail message will be left with a brief message about the study. After five attempts, if study team members are unable to contact an administrator, no further contact will be made. The site will be replaced with the next prioritized site in the sampling frame and the recruitment process will be repeated.

### 2.4. Defining Cases for Phase II (Frontline Staff, Patients, Family Caregivers’ Perspectives)

PAC community partners raised concerns about the impact of the payment policies on certain vulnerable patient populations in light of past evidence that has documented differences in access, care delivery, and quality based on urban–rural status [71,83,84,85,86]. These concerns were reinforced by evidence documenting inequities in COVID-19 cases [87]. In response, Phase II cases are constructed to reflect three categories: (a) urban organizations serving urban populations, (b) urban organizations serving both urban and at least 10% rural populations, and (c) rural organizations serving 80% or greater rural population.

### 2.5. Phase II Sampling Frame and Eligibility

Eligible organizations will have participated in Phase I interviews. Among these Phase I participants, we will target 24 organizations, including 12 SNFs and 12 HHAs. We will recruit four organizations within each case, representing the region where they are located (i.e., Northwest, Midwest, South, West). We will seek variations of representation across patients’ racial and ethnic identities (Table 2). We will prioritize organizations with the lowest representation of White patients, seeking variation in patients identifying as Black, Hispanic, Asian and Pacific Islander, and Native American [82]. Following the methods described in Phase I, we will leverage publicly available data to identify the organizations that meet these criteria.

Eligible frontline staff will include individuals employed by the organization (not temporary agency staff) who are at least 18 years of age or older. We will seek perspectives from multiple staff that have job roles associated with direct patient care, documentation, and/or coding related to the SNF Minimum Data Set (MDS), the HHA Outcome Assessment Information Set (OASIS), and admission and discharge planning. Examples of job titles include admissions and discharge coordinators (e.g., case coordinators, case managers, admissions coordinators), nursing staff (e.g., registered nurse, licensed practical nurse, certified nursing assistant, MDS coordinator), social workers, and rehabilitation staff (e.g., occupational, physical, speech therapy staff).

Eligible patients and family caregivers will include PAC patients receiving care from one of the twenty-four participating SNFs or HHAs. Patients will not be eligible if they are not receiving skilled services, such as respite or home and community-based long-term services and support services. Eligible family caregivers will be the primary contact for the PAC patient (e.g., relative, spouse, partner, neighbor) [88].

### 2.6. Data Collection

Study data will be collected and managed using REDCap electronic data capture tools hosted by the Clinical and Translational Sciences Institute (CTSI) at the University of Pittsburgh [89,90]. REDCap (Research Electronic Data Capture) is a secure, web-based software platform designed to support data capture for research studies, providing (1) an intuitive interface for validated data capture; (2) audit trails for tracking data manipulation and export procedures; (3) automated export procedures for seamless data downloads to common statistical packages; and (4) procedures for data integration and interoperability with external sources. All recruitment-related activities (e.g., sampling frame tracking, efforts to contact potential participants) and data collection, including interview scheduling, capturing participant background information, IRB-approved consent scripts, interview guides, and payment-related activities, will be documented in the project’s REDCap database [89,90].

#### 2.6.1. Interview Guide Development

The study team used the interview guide development process outlined by Kallio et al. (2016), who ensures the research question can be answered through qualitative semi-structured interviews [91]. The team created interview guides informed by the Institute of Medicine framework, previous and emergent evidence (e.g., PAC payment policies, disparities in PAC, and the impact of COVID-19), co-investigator feedback, and cognitive testing of questions.

Phase I interview guides were structured to understand how the environment (i.e., payment reform, COVID-19) impacted the organization (i.e., operations), care team (i.e., staffing), and patient care delivery. The cognitive testing of administrator interview guides involved discussing each question and probing in depth to ensure relevance, flow, clarity, and comprehensiveness. Two HHA experts and one SNF expert who have decades of experience in the respective PAC setting participated in cognitive testing. Feedback from cognitive testing was incorporated into interview guides, and updated documents were shared with co-authors for additional feedback before finalizing and submitting for IRB approval. Refer to Supplemental File S1 for the final version of the Phase I interview guide.

Phase II will use three separate interview guides. The frontline staff interview guide focuses on their experiences within the organization with the new payment policies and the evolution of COVID-19 as it relates to care delivery, work environment, staffing, and infection control policies. Patient and family caregiver interview guides focus on their HHA or SNF care experiences, including met and unmet care needs. Cognitive testing of the interview guides included nine frontline staff (six SNF and three HHA), one patient, and family caregivers (n = 2). Refer to Supplemental File S2 for the Phase II interview guides for staff, Supplemental File S3 for patients, and Supplemental File S4 presents interview questions for family caregivers. We will also re-engage with the administrator to follow up on Phase I interviews by engaging in a member checking session and exploring additional insights the administrator has had since our initial interview.

#### 2.6.2. Background and Sociodemographic Information

At the start of the interview, we will collect demographic and background information on each study participant in order to describe each of the study samples (refer to Supplemental File S5 for details of each variable and specification). Demographic information will capture participants’ self-identified social identities (e.g., gender, race, ethnicity), age, educational attainment, and marital status [92,93,94,95]. SNF/HHA administrators and staff background information will capture key information about their position within the qualifying organization, such as how long they have been employed by the SNF/HHA, job role title, and duration of time in that role [96,97]. Family caregivers will be asked to identify their relationship to the patient (e.g., adult child, spouse/partner), if they live in a shared residence, and whether this is a new role in light of the qualifying acute hospitalization that facilitated the SNF/HHA stay or if this is a long-standing role they have held [98,99,100]. Patients will be asked about their household, the context of their hospitalization, and health insurance coverage [93].

#### 2.6.3. Interviewer Training

To ensure the standardization of interviews per the study protocol, all team members who will be completing interviews will participate in a multimodal training program. Training will include reviewing the study interview manual, participating in a two-hour in-person training session, and then completing three mock interviews for each setting-specific interview guide with a debrief with the project coordinator or principal investigator (PI). The two-hour in-person training session will involve a didactic presentation on the background and purpose of the study, an interactive discussion of the purpose of interviews, and small group interview practice. Only after completing the in-person training session will interviewers engage in the six mock interview sessions. Each of the six interview sessions will last approximately 1.5 h. After all training has been completed and interviewers have transitioned to independent interviews, the first three to five interviews will be reviewed by one study team member within 24 h. This process will allow interviewers to gain confidence with the interview guide, receive additional advice, and ensure compliance with the protocol. Subsequently, every tenth interview will be reviewed by the PI or project coordinator to minimize protocol drift.

### 2.7. Analysis

Rapid qualitative analysis will be used as the analytic approach given the efficacy and efficiency of this approach with large volumes of qualitative data [101]. This approach is well suited to multiple case study designs grounded in frameworks, considering these studies tend to take a more descriptive rather than interpretive approach [101,102,103,104,105,106]. During data collection, the team will engage in data memoing of interviews to inform the development of the data entry matrix [101,107].

Multiple team members will engage in an iterative process in which they immerse themselves in the data and refine the data entry matrix. Through this process, the matrix will be revised to clearly reflect (a) distinct concepts that emerged from interviews and (b) the respective domains of the theoretical framework that informed the structure of the interview guide [106]. Study team members will engage in weekly discussions to examine shared codes and issues with the broad co-investigator team for feedback until a consensus is reached on the codes included in the data entry matrix and corresponding codebook. At this time, the data matrix will be built into REDCap. The matrix will be used for coding all interviews.

Two duplicate REDCap projects will be made for each setting and sample-specific matrix, one for training and a second for independent coding. Once the matrix is built in REDCap, three study team members will develop five training transcripts to be used during coder training, by setting and sample. This entails all three study team members coding the same five transcripts to ensure agreement on the completion of the matrix for each interview. If there is any disagreement among the three coders on any discrete field or narrative field, the team will meet to discuss disagreements and come to a consensus on final responses in the matrix for each of the five transcripts that will be used for training. Consensus for these training transcripts includes 100% agreement on the discrete field response and narrative summary items supporting the discrete field response.

All coders-in-training will go through a 60 min didactic training delivered by one of the project principal investigators (NEL). This session will include an introduction to the methodology, the coding process, REDCap as a data entry platform, the matrix, and a sample-specific codebook. The coder-in-training will then be given access to the training project in REDCap to practice applying the matrix to the training transcripts. Coders-in-training will have to achieve (a) 80% reliability on both discrete fields and (b) consensus on narrative fields for at least three of the five transcripts before being cleared for independent coding [105]. The established expectation of a minimum standard for intercoder reliability aims to limit deviation from the codebook by examining both types of fields [108]. Once a coder-in-training transitions to an independent coder, at least three of the first five transcripts will be coded by a second independent coder. After which, at least one transcript for every ten coded transcripts will be double-coded by a second team member to monitor codebook compliance and limit codebook drift [108]. Finally, in addition to these minimum expectations for a second coder to ensure compliance with the analytic protocol, any independent coder can flag a transcript as needing a second review.

The team of trained coders will follow the three-step process for rapid qualitative analysis [101]. Step one includes each coder’s review and summary of a transcript by domain and category in the REDCap database. Step two will include the reduction, synthesis, and assessment of similarities and differences in the data through the use of completed matrices. Step three will include summary tables in which data are combined and themes are developed. We will explore patterns and differences in themes within and across cases.

Participant background and demographic data will be exported from REDCap and imported into STATA to conduct descriptive analysis [109]. We will characterize each study sample separately for administration, staff, patients, and family caregivers, including the use of means, standard deviations, percentages, or interquartile range. Further, we will leverage our integrated database (previously described in Section 2.2) to characterize SNF and HHA organizations that participate in the study (Supplemental File S6). This database will include variables such as chain status, years certified by CMS as a SNF or HHA, staffing, and aggregate information about the patient populations served.

### 2.8. Scientific Rigour

To ensure methodological rigor and validity, the study team will ensure compliance with the study protocol, which includes triangulation of conceptual model, techniques, and team member perspectives. Throughout all study processes and procedures, we will integrate the reflexivity and authenticity of all members through field notes.

### 2.9. Ethical Considerations

This study protocol was designed to comply with the Declaration of Helsinki and has been approved by the University of Pittsburgh Institutional Review Board (IRB). All methods will comply with this Institution-approved protocol (STUDY20110319) and will be carried out in accordance with relevant guidelines and regulations. Participation in the study protocol is voluntary and can be terminated at any time by participants without repercussions. At the start of each interview, participants will be provided with an oral overview of the study objectives, including possible benefits and risks to participation. Participants are offered a written document that describes the aforementioned script and provides contact information for the principal investigator and the University of Pittsburgh IRB. Signed consent has been waived by the University of Pittsburgh IRB, as the signature is the only identifier for the participant (STUDY20110319).

## 3. Discussion

This study protocol is innovative in that it will be the first study to comprehensively explore payment reform and COVID-19 from multiple perspectives, including administrators, frontline staff, patients, and family caregivers. Capturing their experiences with organizational operations, staffing, and care delivery will provide insight into the state of healthcare in light of these events. A strength of the protocol is its methodologic approach to creating a sampling frame that ensures adequate representation of facilities based on important characteristics, such as location and quality ratings.

Given the complex nature of PAC, this study is necessary to gain a holistic understanding of how organizations navigated these events and their current state, including similarities and differences based on the quality rating of an organization. Staff experiences can help to contextualize how job roles changed, evolved, or remained the same given the new incentives of these payment programs and the impact of COVID-19 on organizational operations. The experiences of patients and family caregivers can provide insights into the new reality of PAC. They can provide information on how they access PAC, including the extent to which they were included in the decision on where to receive PAC after the qualifying acute care hospitalization. We will also explore their experiences with care delivery, including the extent to which patients and family caregivers are engaged in the development of the care plan, goal setting, and discharge planning.

## 4. Limitations

This study is not without limitations. Purposive sampling was chosen to capture a range of SNFs and HHAs in different regions of the country that have differential access to resources, staff, and serve a range of patient populations. Despite these efforts to capture a wide range of organizational perspectives, this study will not be generalizable to the experiences of the more than 15,000 SNFs and 11,400 HHAs in the United States. Further, within each organization, we will be seeking perspectives of key staff who may be able to talk about the impact of the payment policies and pandemic; however, these insights will not be reflective of all staff who work in SNFs and HHAs. Despite these limitations, this study will address a gap in current PAC evidence and lay the foundation for future work to evaluate emergent qualitative themes in a systematic manner in future studies.

## 5. Conclusions

Findings from this study can inform future policy revisions, organizational strategies to promote positive staff and patient outcomes, efforts to navigate the next event that unexpectedly impacts care delivery (e.g., highly contagious condition, natural disaster), and future studies exploring how to improve organizational operations. Moreover, this study protocol is part of a larger mixed methods study that strives to provide evidence addressing the concerns of the PAC community—did the cumulative impact of SNF and HHA payment reform and COVID-19 disproportionately impact vulnerable Medicare beneficiaries?

## Figures and Tables

**Table 1:** Phase I sampling frame.

			Skilled Nursing Facility (n = 150 Maximum Administrators)			Home Health Agency (n = 150 Maximum Administrators)		
			Case 1	Case 2	Case 3	Case 1	Case 2	Case 3
Region			Low	Mod	High	Low	Mod	High
Region 1—Northeast	Urban	For profit	n = 3–5					
		Non-profit/government						
	Rural	For profit						
		Non-profit/government						
Region 2—Midwest	Urban	For profit						
		Non-profit/government						
	Rural	For profit						
		Non-profit/government						
Region 3—South	Urban	For profit						
		Non-profit/ government						
	Rural	For profit						
		Non-profit/government						
Region 4—West	Urban	For profit						
		Non-profit/government						
	Rural	For profit						
		Non-profit/government						
Case-specific sample maximum			n = 50	n = 50	n = 50	n = 50	n = 50	n = 50

Note: For each cell, recruitment will seek a maximum of five administrators with a range of three to five and a maximum of fifty organizations per case.

**Table 2:** Phase II sampling frame.

Region	Case 1	Case 2	Case 3
	Urban-Located Organization-Serving Urban Populations	Urban Located Organization-Serving Combination of Urban and Rural Populations (>10%)	Rural-Located Organization-Serving Rural Population (>80%)
Region 1—Northeast	SNF (n = 1), HHA (n = 1)		
R2—Midwest			
R3—South			
R4—West			
Case-specific sample total	SNF (n = 4), HHA (n = 4)	SNF (n = 4), HHA (n = 4)	SNF (n = 4), HHA (n = 4)

Note: For each cell in the sampling frame, each clinical setting will have one organization represented. Within each organization, recruitment will seek a maximum of twelve staff and no more than six patients and/or family caregivers.

## Data Availability

All the data collection tools, including individual interview guides, focus group guides, and questionnaires, are available in the Supplementary Materials. Since this is a study protocol and not the results of a study, we are not presenting results, so we do not have a database to share in this article.

## Supplementary Materials

The following supporting information can be downloaded at: https://www.mdpi.com/article/10.3390/ijerph20206959/s1, File S1: SNF/HHA Phase I Administrator Interview Guide; File S2: SNF/HHA Phase II Frontline Staff Interview Guide; File S3: SNF/ HHA Patient Interview Guide; File S4: SNF/ HHA Family Caregiver Interview Guide; File S5: Demographic and background information for interviews; File S6: SNF and HHA Organizational Characteristics.
