# Supplementary material for: Implementation of US Post-Acute Care Payment Reform and COVID-19 Policies: Examining Experiences of Health System Leaders, Staff, Patients, and Family Caregivers—A Protocol
Source: Int J Environ Res Public Health. 2023 Oct 23;20(20):6959. doi: 10.3390/ijerph20206959 (PMC10606322; doi:10.3390/ijerph20206959)
Supplement: Supplementary file 1 [file ijerph-20-06959-s001.zip › Supplemental File S1.pdf]

Supplemental File S1. SNF/HHA Phase I Administrator Interview Guide

INTRODUCTION

Hello, my name is ____. I am calling from the University of Pittsburgh about the study "Impact of Post-Acute Care Changes on Access and Outcomes." We had an interview scheduled at this time to discuss how your organization has responded to payment reform in the midst of the global pandemic. Is now still a good time for you to participate in the interview?

Thank you for joining this call to share your experiences. Before we begin, I am going to share some information about our study and your rights as a research participant.

[FOR HHA ADMINISTRATORS]

The purpose of this research study is to learn about the experiences of home health agencies as they navigated PDGM and the COVID-19 pandemic. We will be interviewing approximately 150 home health administrators from all over the country. The interview will take about 45 to 60 minutes to complete.

[FOR SNF ADMINISTRATORS]

The purpose of this research study is to learn about the experiences of nursing home facilities as they navigated PDPM and the COVID-19 pandemic. We will be interviewing approximately 150 nursing home administrators from all over the country. The interview will take about 45 to 60 minutes to complete.

[FOR FACILITATOR: REVIEW INFORMED CONSENT FORM AND ANSWER ANY QUESTIONS]

There is no direct benefit from taking part in this study. Your participation in the study and what you share during the interview will not be disclosed to anyone outside the research team, including your organization or boss. There is a small risk of breach of confidentiality.

To protect your privacy, all personal information will be stored on a secure server here at the University of Pittsburgh separate from the interview data. We will audio record and transcribe your interview to make sure we understand your experiences. During the transcription process any personal information captured on the recording will be deleted. Only the de-identified transcripts will be used for analysis so no information you share will be traced back to you.

Your participation in this study is voluntary. If you do decide to take part in this study, you are free to change your mind and withdraw from the study at any time. If you decide to participate and complete the interview, you will receive a $50 e-gift card in appreciation for your time and insight.

You may contact Dr. Natalie Leland [provide phone number] with any questions, concerns, or complaints about the research or your participation in this study.

If you have any questions about your rights as a research subject or wish to talk to someone other the research team, please call the University of Pittsburgh Human Subjects Protection Advocate [provide phone number].

Do you have any questions before we begin?

Great, I have a few background questions to ask you before we get into the interview, then we will start the interview.

[DEMOGRAPHIC/BACKGROUND QUESTIONS, SEE ADDITIONAL FILE 7]

Thank you for that information. Now we will transition to the interview questions so we can hear your story.

I am going to begin recording. **[START THE AUDIO RECORDING DEVICE]**

The audio tag for this file is **[FOR FACILITATOR: STATE STUDY ID NUMBER]**.

BACKGROUND INFORMATION

[FOR HHA ADMINISTRATORS]

The last two years have brought significant changes with the implementation of the Patient Driven Groupings Model also referred to as PDGM, and the emergence of COVID-19. During this interview we want to hear about your experiences navigating the operations, staffing, and care delivery within your agency for both of these events.

We are interested in hearing about your experiences and will explore three time periods:

- First, in the early days when Centers for Medicare and Medicaid Services first announced that PDGM was going to be implemented
- Then during the initial months after the implementation of PDGM, but before COVID-19.
- And, finally after the emergence of COVID-19 as a federal and state public health emergency.

[FOR SNF ADMINISTRATORS]

The last two years have brought significant changes to the nursing home industry with the implementation of the Patient Drive Payment Model, also referred to as PDPM, and the emergence of COVID-19. During this interview we want to hear about your experiences navigating the operations, staffing, and care delivery within your facility for both of these events.

We are interested in hearing about your experiences and will explore three time periods:

- First, in the early days when Centers for Medicare and Medicaid Services first announced that PDPM was going to be implemented
- Then during the initial months after the implementation of PDPM, but before COVID-19.
- And, finally after the emergence of COVID-19 as a federal and state public health emergency.

INTERVIEW QUESTIONS

[FOR HHA ADMINISTRATORS]

1. We will start by talking about the policy itself. How did you learn about PDGM? *Probes: information/resources (education provided by employer, National Association materials,*

webinars)

2. How did this knowledge inform your approach to payment reform?

3. Now we want to understand the impact of PDGM on home health operations: How have your agency's operations been impacted by payment reform? *Probes: admission practices (change in patient population/case mix (diagnosis, care needs, insurance type), change in referral sources (i.e., community vs. post-acute care), changes in specialty care services, communications with discharging facilities (e.g., protocols with preferred providers)), changes in length of stay, changes in financial operations as a result of PDGM reimbursement, changes in information systems (e.g., documentation and reporting practices), discharge practices (communication with receiving organization/primary care provider, coordination with primary care provider)*

4. Were any changes you described made before the start of PDGM? Or, have all of the changes you described occurred after PDGM was implemented? *We will use answers from #3 to frame the question. For example, "You mention your organization implemented a new documentation system, was this change made before the start of PDGM? Or, have all of the changes you described occurred after PDGM was implemented?"*

5. Have there been any further modifications since the policy was implemented?

6. Since COVID emerged, have you had to make further modifications or changes? How? *Probes: admissions (change in census (if decrease, was decrease due to: pause in elective surgeries from hospital, limited access to PPE, and/or patients refusing home health services; if increase, was increase due to changes in hospital referral practices, such as patients funneled to home health agencies versus skilled nursing facilities due to outbreaks)), PPE (e.g., access to equipment, costs of the equipment, other equipment-related charges), financial constraints (adequacy of infection control resources, adequacy of infection control practices, change in census vs. high cost of PPE, COVID Relief Act or CARES Act Support), policies (focus on infection control, communication between staff re: COVID-19, communication with family (e.g., use of technology), impact on patient needs)*

7. How did vaccination availability influence operations at your agency?

8. How has your agency approached therapy staffing under PDGM? *Probes: in-house vs. Contract therapy (if kept contract therapy company, were expectations of contract revised due to PDGM? How?), reduction in staffing (i.e.., layoffs, furloughs), reductions in staff pay (i.e., pay freezes or cuts), distribution of rehab staff (e.g., use of registered therapists vs. aides/assistants, use of physical therapy, use of occupational therapy, use of speech language pathology), caseload among therapy providers (i.e., number of patients seen per therapist/day), expectations for productivity*

9. How has your agency approached nursing staffing under PDGM? *Probes: given the emphasis on documentation, were there any changes in roles or new hires among nursing to meet needs of the payment policy, productivity standards for nursing, nurse staff changes (i.e., use of registered nurses vs. Licensed practical nurses, changes in terms of distribution of staff, changing roles to meet emerging needs of the payment policy), use of restorative services to foster carryover from therapy, use of home health aides*

10. Were any changes you described made before PDGM was implemented? Or, have all of the changes you described occurred after PDGM started? *We will use answers from #8 and 9 to frame the question. For example, "You mention your organization switched from using contract therapists to using only in-house therapists; was this change made before PDGM was implemented? Or did this occur after PDGM started?"*

11. Have there been any further modifications since the policy was implemented?

12. Since COVID emerged, have you had to make further modifications or changes? How? *Probes: turnover, reallocation of staff to address emerging critical needs (e.g., redistribution of workflow, work responsibilities), burnout/coping strategies/mental health of staff, prioritization/categorization of who is essential staff, changes in wage scale to recruit or maintain staff*

13. How did vaccine implementation influence staffing changes? *Probes: prior to the CMS vaccine mandate in August 2021, after the CMS vaccine mandate in August 2021*

14. How did PDGM influence care delivery policies and practices at your agency? *Probes: reimagined policies related to therapy services (e.g., who gets therapy (case mix for short-stay, Medicare Part B), maintenance therapy, when is therapy provided, how much therapy is provided, were there guiding principles that informed decisions about delivery of therapy services), reimagined approaches to interdisciplinary care delivery, restorative nursing, resources (if case mix has changes: are there resource opportunities? Are there additional needs? If case mix is the same, are there resource opportunities? Are there additional needs?), prioritizing quality initiatives, patient populations that are at risk for poor outcomes, barriers and facilitators to enhancing/maintaining quality, staff training (i.e., response to new payment models (e.g., how to change 20 years of the Prospective Payment System behaviors for new billing), response to emerging care needs)*

15. Were any changes you described made before PDGM was implemented? Or, have all of the changes you described occurred after PDGM started? *We will use answers from #14 to frame the question. For example, "You mention interdisciplinary approaches to care delivery was increased through weekly collaborative meetings; was this change made before PDGM was implemented? Or did this occur after PDGM started?"*

16. Have there been any further modifications since the policy was implemented?

17. Since COVID emerged, have you had to make further modifications or changes? How? *Probes: infection control practices, impact on care quality, impact on patient safety, barriers & facilitators to enhancing/maintaining quality (e.g., finances, staff training, staff resources), use of telehealth (e.g., challenges, opportunities, when to use & for which patients)*

18. How did vaccination availability influence care delivery? *Probes: allocation of services, enhancing/maintaining quality*

[FOR SNF ADMINISTRATORS]

1. We will start by talking about the policy itself. How did you learn about PDGM? *Probes: information/resources (education provided by your facility, the organization's corporate office which your facility is affiliated with, your contracted rehab provider, National Association materials, State Association materials, webinars)*

2. How did that knowledge inform your organizational policies to transition to the new payment model?

3. How did that knowledge inform your organizational practices to transition to the new payment model?

4. Now we want to understand the impact of PDPM on nursing home operations. How have your operations been impacted by PDPM? *Probes: pre-admission practices, admission practices (e.g., change in patient populations/case mix (diagnosis, dementia/cognitive impairment, care needs, insurance types), changes in referral sources (e.g., limiting/changing partnerships with hospitals), partnerships with discharging facilities (e.g., preferred provider network, communication practices, formalized relationships), refocus/promote specialty care (e.g., vent units)), changes in length of stay, discharge practices (e.g., changes in where patients are being discharged to, coordination with receiving provider (primary care physician, home health providers)), financial impact of the payment policy on facility operations in first 6 months of PDPM, revenue, changes in information systems (e.g., changes in documentation fields, reporting practices, MDS artificial intelligence to assist with coding)*

5. Of the changes that were made in your facility, were any of them implemented as part of a corporate strategy (i.e., if facility was part of a chain)? Or were these changes implemented by the facility?

6. What, if any, of those changes in nursing home operations were made in anticipation of PDPM? What, if any, of those changes in nursing home operations were made after the start of PDPM, prior to the impact of COVID within your region? *We will use answers from #4 to frame the question. For example, "You mention your organization implemented a new documentation system, was this change made before the start of PDPM? Or, have all of the changes you described occurred after PDPM was implemented?"*

7. After the emergence of COVID-19 within your region, what was the cumulative impact of PDPM and the pandemic on nursing home operations? *Probes: change in census (e.g., "For example, early in the pandemic when some hospitals suspended elective surgeries?"; "Later into spring and/or summer of 2020, as the pandemic progressed were there other census changes?"; "Did your organization establish a COVID-19 unit/floor?"; "Were there other census changes?"), use of waivers (e.g., such as the waiver that requires a 3-day acute hospital stay to access post-acute care services, telehealth waiver), PPE (e.g., access ("How did you get your PPE?" (e.g., donations from World Health Organization, purchase on open market)), costs of Personal Protective Equipment, other challenges related to PPE), access to COVID-19 testing for staff/residents/consultants/visitors, financial constraints associated with the COVID-19 public health emergency (e.g., adequacy of infection control resources (such as Personal Protective Equipment), adequacy of infection control practices (such as the need to deliver additional training), COVID Economic Relief, CARES Act support (if yes to CARES Act: "Did you use it?", "Did you find it beneficial?", "Did you return it and why?"), policies focus on infection control, communication strategies about COVID-19 between facility leadership and staff/patients/family (e.g., use of technology to share information (e.g., zoom, e-newsletter, texting), strategies for connecting residents/patients and family, other forms of communication), impact on resident quality of life*

8. How did vaccine implementation influence these operations? *Probes: prior to the CMS vaccine mandate in August 2021, after the CMS vaccine mandate in August 2021, does your state have a vaccine mandate (if yes: "When was that implemented?"; "How did that state policy impact your operations?")*

9. I am curious about the impact of PDPM on nursing home staffing. How has your organization approached therapy staffing under PDPM? *Probes: prior to PDPM, did you have in-house or contract therapy?, did your facility approach therapy staffing differently due to PDPM? (e.g., change staffing approach from in-house to contract or contract to in-house therapy) if kept the contract therapy company, was the agreement with the company revised due to PDPM? How?, if kept in-house staff, did the approach to staffing change? How?, managing change in productivity metrics (e.g., staff caseload expectations, staff productivity, under the old system productivity was measured by billable minutes of therapy, how was productivity reimagined under PDPM), reduction in staffing (e.g., layoffs, furloughs), reduction in pay (e.g., pay cuts, pay freezes), change in the distribution of staff (e.g., use of registered therapists vs. assistants/aides, use of physical therapy, use of occupational therapy, use of speech language pathology)*

10. How has your organization approached nursing staffing under PDPM? *Probes: given the emphasis on documentation, were there changes in roles among current staff to meet needs of the payment policy, new hires among nursing to meet needs of the payment policy (e.g., such as changes in admissions coordinator), were there staff changes as it relates to distribution of registered nurses vs. licensed practical nurse, changes as it relates to changing roles of existing nursing staffing given emerging needs of the policy, changes as it relates to weekday vs. Weekend coverage, use of restorative services to*

foster carryover from therapy (e.g., training CNAs to become rehabilitation nursing assistants), use of therapy aides, productivity standards for nursing

11. How has your organization approached physician staffing under PDPM? *Probes: PDPM places a big emphasis on diagnoses which have to be based on written documentation by provider, did they make any changes to meet this need (e.g., role of medical director, privileging of providers)*

12. How has your organization approached pharmacist staffing under PDPM?

13. Were there any other policies related to staffing with PDPM?

14. What, if any, of those changes in nursing home staffing were made in anticipation of PDPM? What, if any of those changes in nursing home staffing were made after the start of PDPM, prior to the impact of COVID within your region? *We will use answers from #9-13 to frame the question. For example, "You mention your organization switched from using contract therapists to using only in-house therapists; was this change made before PDPM was implemented? Or did this occur after PDPM started?"*

15. After the emergence of COVID-19 within your region, what was the cumulative impact of PDPM and the pandemic on nursing home staffing? *Probes: turnover, reallocation of staff to address emerging critical needs (e.g., patient care, training backup staff to execute pivotal MDS reporting activities (e.g., PHQ-9, BIMS, MDS), restrictions on contract therapists not being allowed in the facility), coverage of positive staff cases, burnout/coping strategies/mental health of staff, prioritization/categorization of who is essential staff, changes in wage scale to recruit or maintain staff*

16. How did vaccine implementation influence staffing changes (e.g., rehabilitation, CNAs)? *Probes: Prior to the CMS vaccine mandate in August 2021, after the CMS vaccine mandate in August 2021, "Does your state have a vaccine mandate?" (if yes: "When was that implemented?", "How did that state policy impact staffing?")*

17. I would like to hear about your perceptions of the impact of PDPM on nursing home care delivery. How did payment reform influence care delivery policies and practices at your facility? *Probes: reimagined policies related to therapy services (e.g., length of stay, initiation of services, service delivery), reimagined practices related to therapy services (e.g., who gets therapy (e.g., case mix for short-stay, changes in care delivery for Medicare Part B for long-stay residents, change in delivery of maintenance therapy), what therapy patients receive (i.e., physical therapy, occupational therapy, speech language pathology), criteria for continuing therapy (e.g., how are/were decisions made on who gets therapy services (i.e., physical therapy, occupational therapy, speech language pathology)), criteria for discontinuing therapy, were there guiding principles for therapy service delivery (when?, how much?, in what form? (e.g., 1:1 treatment sessions, concurrent therapy, group therapy)), changes in the interdisciplinary approach to care delivery, changes in use of restorative nursing, prioritizing quality initiatives (I.e., how were the Centers for Medicare and Medicaid quality measures prioritized?, what*

were the barriers to enhancing/maintaining quality (e.g., fall prevention initiatives, readmissions, managing resident depression), what were the facilitators to enhancing/maintaining quality (e.g., fall prevention initiatives, readmissions, managing resident depression)), staff training (i.e., to prepare for new payment models (e.g., such as how to change provider behaviors based on 20 years of the Prospective Payment System, how to inform provider behaviors to address new billing requirements), to prepare for new approaches to care (e.g., emphasis on interdisciplinary care coordination))

18. What, if any, of those changes in nursing home care delivery policies and practices were made in anticipation of PDPM? What, if any, of those changes in nursing home care delivery were made after the start of PDPM, prior to the impact of COVID within your region? *We will use answers from #17 to frame the question. For example, "You mention interdisciplinary approaches to care delivery were increased through weekly collaborative meetings; was this change made before PDPM was implemented? Or did this occur after PDPM started?"*

19. After the emergence of COVID-19 within your region, what was the cumulative impact of PDGM and the pandemic on nursing home care delivery? *Probes: prioritization of care quality and/or infection control, "How did you navigate evolving requirements for managing COVID-19 at the local, regional, and national level?" (e.g., organizational leadership calls, stakeholder calls, national industry/association informational calls), barriers to care quality given the mandated heightened infection control policies and public health emergency (e.g., finances, staff training, staff resources), facilitators to care quality given the mandated heightened infection control policies and public health emergency (e.g., finances, staff training, staff resources), telehealth (e.g., opportunities for use? Challenges? When to use? For which patients?), with staff turnover, what strategies were used to equip new staff with an understanding of COVID-19 policies and PDPM expectations?*

20. How did vaccine implementation influence care delivery? *Probes: allocation of services, addressing care quality (e.g., enhancing/maintaining)*

REFLECTION QUESTIONS

[FOR HHA ADMINISTRATORS]

Thinking about lessons you have learned over the last two years with the implementation of PDGM and the global pandemic:

1. Did your Agency participate in value-based initiatives prior to PDGM? If so, did your involvement in past initiatives inform your response to PDGM? If yes, how?
2. Were there any lessons your organization learned from watching the nursing home industry go through PDPM three months before you?
3. What advice would you give to yourself or your peers, if you could go back in time and provide some sage advice?

4. What would you want policy makers to know about the experiences of home health agencies over the last two years?
5. What would you want the public to know?
6. Is there anything else you would like to share that I have not asked you?

[FOR SNF ADMINISTRATORS]

Thinking about lessons you have learned over the last two years with the implementation of PDPM and the global pandemic, if you could go back in time and provide some sage advice:

1. What would you want the public to know?
2. What advice would you give to yourself or your peers?
3. What would you want policymakers to know?
4. Is there anything else you would like to share that I have not asked you?
