# Supplementary material for: Implementation of US Post-Acute Care Payment Reform and COVID-19 Policies: Examining Experiences of Health System Leaders, Staff, Patients, and Family Caregivers—A Protocol
Source: Int J Environ Res Public Health. 2023 Oct 23;20(20):6959. doi: 10.3390/ijerph20206959 (PMC10606322; doi:10.3390/ijerph20206959)
Supplement: Supplementary file 1 [file ijerph-20-06959-s001.zip › Supplemental File S2.pdf]

Supplemental File S2. SNF/HHA Phase II Frontline Staff Interview Guide

INTRODUCTION

Hello, my name is ____. Thank you for joining this call to share your experiences. Before we get started, I am going to take a few minutes to read some information that is required by my university. If you have any questions about this material or would like any of the information to be repeated, please do not hesitate to ask. Then we can get into the interview.

I am going to share some information about our study and your rights as a research participant. The purpose of this research study is to learn about the experiences of home health/skilled nursing facility staff as they navigated payment reform and COVID-19. We will be interviewing home health and skilled nursing staff from all over the country. The interview will take about 45 minutes to complete.

[FOR FACILITATOR: REVIEW INFORMED CONSENT FORM AND ANSWER ANY QUESTIONS]

There is no direct benefit from taking part in this study. Your participation in the study and what you share during the interview will not be disclosed to anyone outside the research team, including your organization or boss. There is a small risk of breach of confidentiality.

To protect your privacy, all personal information will be stored on a secure server here at the University of Pittsburgh separate from the interview data. We will audio record and transcribe your interview to make sure we understand your experiences. During the transcription process any personal information captured on the recording will be deleted. Only the de-identified transcripts will be used for analysis so no information you share will be traced back to you.

Your participation in this study is voluntary. If you do decide to take part in this study, you are free to change your mind and withdraw from the study at any time. If you decide to participate and complete the interview, you will receive a $50 e-gift card in appreciation for your time and insight.

You may contact Dr. Natalie Leland at (412)383-3405 with any questions, concerns, or complaints about the research or your participation in this study. If you have any questions about your rights as a research subject or wish to talk to someone other the research team, please call the University of Pittsburgh Human Subjects Protection Advocate toll-free at 866-212-2668.

Do you have any questions about this overview?

Great, I have a few background questions to ask you before we get into the interview, then we will start the interview.

[DEMOGRAPHIC/BACKGROUND QUESTIONS, SEE ADDITIONAL FILE 7]

Thank you for that information. Now we will transition to the interview questions so we can hear your story.

I am going to begin recording. **[START THE AUDIO RECORDING DEVICE]**

The audio tag for this file is **[FOR FACILITATOR: STATE STUDY ID NUMBER]**.

BACKGROUND INFORMATION

During this interview we want to hear your story to understand your experiences. The last few years have brought significant changes to skilled nursing and home health. First, in October 2019 for nursing facilities and in January 2020 to home health, changes were made to the way Medicare reimbursed health care services. Soon after, in early 2020, the COVID-19 pandemic emerged. We'll ask about your experiences from that time until now.
Do you have any questions before we begin?

INTERVIEW QUESTIONS

1. How did you learn about [PDPM/PDGM]? *Probes: preparation from employer, trainings and resources (easily understandable, useful).*

2. Tell me about your current post-acute care patients. *Probes: health/medical status (diagnoses, medical complexity (e.g., wounds, ventilators, multiple comorbid conditions), dementia/Alzheimer's); communities where patients are from (e.g., insurance status (Medicare, Medicaid, dual eligible, managed care, private insurance), economic status, race and ethnicity); challenges patients face related to discharge planning (e.g., health literacy (knowledge about their health, ability to navigate healthcare system), access to resources, social supports (families and caregivers)); similarities and differences compared to patients prior to PDPM/PDGM or after the emergence of COVID-19.*

3. We want to understand your experiences since PDPM was implemented in October 2019, but prior to the emergence of COVID-19 in March 2020. Can you recall your experience with patient care at that time? *Probes: obstacles related to PDPM; changes in care delivery related to PDPM.*

4. Now, I would like to learn a little more about your day-to day responsibilities. Tell me about what a typical day is like for you. *Probes: responsibilities since the implementation of PDPM; changes in care for rehab post-acute care patients (e.g., intensity of frequency of services (duration, difficulty of interventions); caseload; length of stay; use of telehealth; shifts in use of resources (reallocation of clinical practices between disciplines, for example, walking programs with a nursing aide); productivity standards (implicit and explicit); approach to family and caregiver training); changes to interdisciplinary communication (e.g., team meetings, communication, and coordination; documentation expectations and practices; benefits of PDPM related to day-to-day job); describe job since the emergence of COVID (roles and responsibilities; patient care; interdisciplinary operations; documentation practices.)*

5. Do you think that patient outcomes changed because of PDPM? *Probes: positive changes in patient outcomes or challenges from PDPM; positive changes in patient outcomes or challenges from COVID*.

REFLECTION QUESTIONS

When you think back over this period, with the combination of PDGM/PDPM and COVID-19:

1. What is a work success that stands out to you?
2. What is a work challenge that stands out?
3. Knowing what you know now, what advice would you want to give to yourself or your peers?
4. What would you want the following groups to know about your experience with PDGM/PDPM and COVID-19? *Probes: organizational leadership, patients and caregivers, policymakers, public/community*.
