# Supplementary material for: Implementation of US Post-Acute Care Payment Reform and COVID-19 Policies: Examining Experiences of Health System Leaders, Staff, Patients, and Family Caregivers—A Protocol
Source: Int J Environ Res Public Health. 2023 Oct 23;20(20):6959. doi: 10.3390/ijerph20206959 (PMC10606322; doi:10.3390/ijerph20206959)
Supplement: Supplementary file 1 [file ijerph-20-06959-s001.zip › Supplemental File S3.pdf]

Supplemental File S3. SNF/ HHA Patient Interview Guide

INTRODUCTION

Hello, my name is ____. Thank you for joining this call to share your experiences. Before we get started, I am going to take a few minutes to read some information that is required by my university. If you have any questions about this material or would like any of the information to be repeated, please do not hesitate to ask. Then we can get into the interview.

I am going to share some information about our study and your rights as a research participant. The purpose of this research study is to learn about the experiences of home health/skilled nursing facility patients as they navigated their post-acute care stay. We will be interviewing home health and skilled nursing patients from all over the country. The interview will take about 45 minutes to complete.

[FOR FACILITATOR: REVIEW INFORMED CONSENT FORM AND ANSWER ANY QUESTIONS]

There is no direct benefit from taking part in this study. Your participation in the study and what you share during the interview will not be disclosed to anyone outside the research team, including your organization or boss. There is a small risk of breach of confidentiality.

To protect your privacy, all personal information will be stored on a secure server here at the University of Pittsburgh separate from the interview data. We will audio record and transcribe your interview to make sure we understand your experiences. During the transcription process any personal information captured on the recording will be deleted. Only the de-identified transcripts will be used for analysis so no information you share will be traced back to you.

Your participation in this study is voluntary. If you do decide to take part in this study, you are free to change your mind and withdraw from the study at any time. If you decide to participate and complete the interview, you will receive a $50 e-gift card in appreciation for your time and insight.

You may contact Dr. Natalie Leland with any questions, concerns, or complaints about the research or your participation in this study [provide phone number].

If you have any questions about your rights as a research subject or wish to talk to someone other the research team, please call the University of Pittsburgh Human Subjects Protection Advocate [provide phone number].

Do you have any questions about this overview?

Great, I have a few background questions to ask you before we get into the interview, then we will start the interview.

[DEMOGRAPHIC/BACKGROUND QUESTIONS, SEE ADDITIONAL FILE 7]

Thank you for that information. Now we will transition to the interview questions so we can hear your story.

I am going to begin recording. **[START THE AUDIO RECORDING DEVICE]**

The audio tag for this file is **[FOR FACILITATOR: STATE STUDY ID NUMBER]**.

BACKGROUND INFORMATION

During this interview we want to hear your story to understand your experiences.

Do you have any questions before we begin?

PATIENT INTERVIEW QUESTIONS

[FOR SNF PATIENTS]

First, we would like to hear about how the decision was made for you to be discharged from the hospital to the SNF.

1. Who was involved in making the decision to go to the SNF?
2. How was it chosen? *Probes: factors influencing this decision (e.g., financial, hospital referral or recommendation, location of services, recommendation by family/friends, family/friends received care here, availability of services/accepting admissions); prior wishes for where to be discharged.*
3. Walk me through what happened, starting from when the decision was made for you to go to the SNF, to when you were admitted to the SNF. *Probes: positive experiences with move from hospital to SNF (e.g., communication with hospital, communication with SNF, access to information/ questions answered); role of COVID; challenges; ways to improve experience.*

Now we would like to hear more about the SNF where you were a patient.

4. What did you hope to be able to do when you left the SNF?
5. Please tell me about your experience as a patient there. *Probes: positive aspects of care, challenges, ways to improve experience, impact of COVID, treatment from staff/providers, (if previous SNF stay) comparison with past experience(s).*
6. Did you receive occupational, physical, or speech therapy services? *We will use answers from background questions to frame question. If previously mentioned therapy, for example, "I heard you mention ___ therapy, did you also receive ___ (e.g., occupational, physical, speech)?" Probes: positive experiences, challenges, ways to improve experience.*

[FOR HHA PATIENTS]

First, we would like to hear about how the decision was made for you to be discharged home from the hospital to receive care from the HHA.

1. Who was involved in making the decision to receive care from the HHA?
2. How was it chosen? *Probes: factors influencing this decision (e.g., financial, hospital referral or recommendation, location of services, recommendation by family/friends, family/friends received care here, availability of services/accepting admissions); prior wishes for where to be discharged.*
3. Walk me through what happened, starting from when the decision was made for you to go home with home health care, to the first home care visit. *Probes: positive experiences with move from hospital to home (e.g., communication with hospital, communication with HHA, access to information/ questions answered); role of COVID; challenges; ways to improve experience.*

Now we would like to hear more about the HHA.

4. What did you hope to be able to do when you were discharged from the HHA?
5. Please tell me about your experience receiving care from the HHA. *Probes: positive aspects of care, challenges, ways to improve experience, impact of COVID, treatment from staff/providers, (if previous HHA care) comparison with past experience(s).*
6. Did you receive occupational, physical, or speech therapy services? *We will use answers from background questions to frame question. If previously mentioned therapy, for example, "I heard you mention ___ therapy, did you also receive ___ (e.g., occupational, physical, speech)?" Probes: positive experiences, challenges, ways to improve experience.*

REFLECTION QUESTIONS

[FOR SNF PATIENTS]

Thank you for sharing about your recent experiences with the SNF.

1. If you could go back in time, what advice would you give yourself before entering the SNF?
2. What would you want to tell the SNF?
3. Is there anything else you would like to say about your recent care there?

[FOR HHA PATIENTS]

Thank you for sharing about your recent experiences with the HHA

1. If you could go back in time, what advice would you give yourself before starting to use the HHA?
2. What would you want to tell the HHA?
3. Is there anything else you would like to say about your recent care with them?
