# Supplementary material for: Implementation of US Post-Acute Care Payment Reform and COVID-19 Policies: Examining Experiences of Health System Leaders, Staff, Patients, and Family Caregivers—A Protocol
Source: Int J Environ Res Public Health. 2023 Oct 23;20(20):6959. doi: 10.3390/ijerph20206959 (PMC10606322; doi:10.3390/ijerph20206959)
Supplement: Supplementary file 1 [file ijerph-20-06959-s001.zip › Supplemental File S4.pdf]

Supplemental File S4. SNF/ HHA Family Caregiver Interview Guide

INTRODUCTION

Hello, my name is ____. Thank you for joining this call to share your experiences. Before we get started, I am going to take a few minutes to read some information that is required by my university. If you have any questions about this material or would like any of the information to be repeated, please do not hesitate to ask. Then we can get into the interview.

I am going to share some information about our study and your rights as a research participant. The purpose of this research study is to learn about the experiences of home health/skilled nursing facility patients as they navigated their post-acute care stay. We will be interviewing home health and skilled nursing patients from all over the country. The interview will take about 45 minutes to complete.

[FOR FACILITATOR: REVIEW INFORMED CONSENT FORM AND ANSWER ANY QUESTIONS]

There is no direct benefit from taking part in this study. Your participation in the study and what you share during the interview will not be disclosed to anyone outside the research team, including your organization or boss. There is a small risk of breach of confidentiality.

To protect your privacy, all personal information will be stored on a secure server here at the University of Pittsburgh separate from the interview data. We will audio record and transcribe your interview to make sure we understand your experiences. During the transcription process any personal information captured on the recording will be deleted. Only the de-identified transcripts will be used for analysis so no information you share will be traced back to you.

Your participation in this study is voluntary. If you do decide to take part in this study, you are free to change your mind and withdraw from the study at any time. If you decide to participate and complete the interview, you will receive a $50 e-gift card in appreciation for your time and insight.

You may contact Dr. Natalie Leland [provide phone number] with any questions, concerns, or complaints about the research or your participation in this study. If you have any questions about your rights as a research subject or wish to talk to someone other the research team, please call the University of Pittsburgh Human Subjects Protection Advocate [provide phone number].

Do you have any questions about this overview?

Great, I have a few background questions to ask you before we get into the interview, then we will start the interview.

[DEMOGRAPHIC/BACKGROUND QUESTIONS, SEE ADDITIONAL FILE 7]

Thank you for that information. Now we will transition to the interview questions so we can hear your story.

I am going to begin recording. **[START THE AUDIO RECORDING DEVICE]**

The audio tag for this file is **[FOR FACILITATOR: STATE STUDY ID NUMBER]**.

BACKGROUND INFORMATION

During this interview we want to hear your story to understand your experiences.

Do you have any questions before we begin?

FAMILY/CAREGIVER INTERVIEW QUESTIONS

[FOR SNF FAMILY/CAREGIVERS]

First, we would like to hear about how the decision was made for the patient to be discharged from the hospital to the SNF.

1. Who was involved in making the decision to go to the SNF?
2. How was it chosen? *Probes: factors influencing this decision (e.g., financial, hospital referral or recommendation, location of services, recommendation by family/friends, family/friends received care here, availability of services/accepting admissions); prior wishes for where to be discharged.*
3. Walk me through what happened, starting from when the decision was made to go to the SNF, to when they were admitted to the SNF. *Probes: role and responsibilities in the transition, positive experiences with move from hospital to SNF (e.g., communication with hospital, communication with SNF, access to information/ questions answered); role of COVID; challenges; ways to improve experience.*

Now we would like to hear more about the SNF.

4. Please tell me about your experiences as a caregiver supporting the patient while they were receiving care at the SNF. *Probes: prior experience in caregiving role supporting the patient (if prior experience, past responsibilities, changes in role; if no prior experience, describe new role as the patient's caregiver).*
5. Please tell me about the care at the SNF. *Probes: positive aspects of care, challenges, ways to improve, impact of COVID, inclusion in decision and care by care team (sought out as resource, included in care plan meetings), provided necessary information by healthcare team, provided education or training from care team (medication, therapy), treatment from staff/providers, (if patient had previous SNF stay) comparison with past experience(s).*
6. Did the patient receive occupational, physical, or speech therapy services? *We will use answers from background questions to frame question. If previously mentioned therapy, for example, "I heard you mention ___ therapy, did they also receive ___ (e.g., occupational, physical, speech)?" Probes: inclusion in therapy sessions (if yes, describe experience (e.g., positive experiences, challenges, education or training to prepare for*

discharge (feel prepared, feel needed more); if no, would like to have been included, how could have been facilitated).

[FOR HHA FAMILY/CAREGIVERS]

First, we would like to hear about how the decision was made for the patient to be discharged home from the hospital to receive care from the HHA.

1. Who was involved in making the decision to receive care from the HHA?
2. How was it chosen? *Probes: factors influencing this decision (e.g., financial, hospital referral or recommendation, location of services, recommendation by family/friends, family/friends received care here, availability of services/accepting admissions); prior wishes for where to be discharged.*
3. Walk me through what happened, starting from when the decision was made to go home with home health care, to the first home care visit. *Probes: role and responsibilities in the transition, positive experiences with move from hospital to home (e.g., communication with hospital, communication with HHA, access to information/ questions answered); role of COVID; challenges; ways to improve experience.*

Now we would like to hear more about the HHA.

4. Please tell me about your experiences as a caregiver supporting the patient while they were receiving care at home from the HHA. *Probes: prior experience in caregiving role supporting the patient (if prior experience, past responsibilities, changes in role; if no prior experience, describe new role as the patient's caregiver).*
5. Please tell me about the care from the HHA. *Probes: positive aspects of care, challenges, ways to improve, impact of COVID, inclusion in decision and care by care team (sought out as resource, included in care plan meetings), provided necessary information by healthcare team, provided education or training from care team (medication, therapy), treatment from staff/providers, (if patient had previous HHA care) comparison with past experience(s).*
6. Did the patient receive occupational, physical, or speech therapy services? *We will use answers from background questions to frame question. If previously mentioned therapy, for example, "I heard you mention ___ therapy, did they also receive ___ (e.g., occupational, physical, speech)?" Probes: inclusion in therapy sessions (if yes, describe experience (e.g., positive experiences, challenges, education or training to prepare for discharge (feel prepared, feel needed more); if no, would like to have been included, how could have been facilitated).*

REFLECTION QUESTIONS

[FOR SNF FAMILY/CAREGIVERS]

Thank you for sharing about your recent experiences with the SNF.

1. If you could go back in time, what advice would you give yourself before the patient entered the SNF? What advice would you give the patient?
2. What would you want to tell the SNF?
3. Is there anything else you would like to share about the patient's recent care there?

[FOR HHA FAMILY/CAREGIVERS]

Thank you for sharing about your recent experiences with the HHA

1. If you could go back in time, what advice would you give yourself before the patient started to use the HHA? What advice would you give the patient?
2. What would you want to tell the HHA?
3. Is there anything else you would like to share about the patient's recent care with them?
