# Supplementary material for: Implementation of US Post-Acute Care Payment Reform and COVID-19 Policies: Examining Experiences of Health System Leaders, Staff, Patients, and Family Caregivers—A Protocol
Source: Int J Environ Res Public Health. 2023 Oct 23;20(20):6959. doi: 10.3390/ijerph20206959 (PMC10606322; doi:10.3390/ijerph20206959)
Supplement: Supplementary file 1 [file ijerph-20-06959-s001.zip › Supplemental File S5.pdf]

**Supplemental File S5.** Background and Demographic information for interviews

| Variable name | Questions | Response options | Interview (A, S, P, F) |
|---|---|---|---|
| **Demographics** | | | |
| Age | What is your age (in years)? | • *[insert number in years]* | A, S, P, F |
| Race | How would you best describe your race? | • American Indian or Native American<br>• Asian<br>• Black or African American<br>• Native Hawaiian or Other Pacific Islander<br>• White<br>• Multiracial<br>• Other *[insert open text box]*<br>• Prefer not to say | A, S, P, F |
| Ethnicity | Would you describe yourself as Hispanic or Latino? | • Hispanic or Latino<br>• Not Hispanic or Latino<br>• Prefer not to say | A, S, P, F |
| Gender | How would you describe your gender? | • Female<br>• Male<br>• Transgender woman<br>• Transgender man<br>• Non-binary<br>• Other *[insert open text box]*<br>• Prefer not to say | A, S, P, F |
| Education | What is the highest degree or level of school you have completed? | • Less than high school<br>• High school graduate, diploma or the equivalent (for example: GED)<br>• Trade/technical/ vocational training<br>• Associate degree<br>• Bachelor's degree<br>• Master's degree<br>• Doctorate degree<br>• Other *[insert open text box]*<br>• Prefer not to say | A, S, P, F |
| Marital status | How would you describe your marital status? | • Married<br>• Living with a partner<br>• Separated | P, F |

| | | | |
|---|---|---|---|
| | | • Divorced<br>• Widowed<br>• Never married<br>• Prefer not to say | |
| Relationship to Patient | What is your relationship to *[name of Patient]*? | • Spouse<br>• Parent<br>• Child<br>• Other family<br>• Friend<br>• Other *[insert open text box]*<br>• Prefer not to say | F |
| Shared Residence | Before they were hospitalized, did you and the *[name of Patient]* live in the same home? | • Yes<br>• No<br><br>• *[If no]*: Did you live nearby?<br>  ○ Less than 1 hour away<br>  ○ Between 1 hour and 3 hours away<br>  ○ More than 3 hours away<br>  ○ Unsure<br>  ○ Prefer not to say | F |
| Household makeup | How many people lived in your home, including yourself, at the time *[you/they]* were hospitalized? | • *[insert number]*<br>• Prefer not to say | P, F |
| Zip code | What was your ZIP code at the time *[you/they]* were hospitalized? | • *[insert number]*<br>• Prefer not to say | P, F |
| Qualifying hospitalization and healthcare utilization | | | |
| Reason for Hospitalization | Can you tell me why *[you were/ [name of Patient] was]* hospitalized? | • *[insert text]* | P, F |
| Place of Hospitalization | Where were *[you/they]* hospitalized? | • *[insert text]* | P, F |
| Length of Hospitalization | For how many days were *[you/they]* in the hospital? | • *[insert number]* | P, F |
| Health insurance | What kinds of health insurance or health care coverage *[do* | • Private health insurance<br>• Medicare | P, F |

| | | | |
|---|---|---|---|
| | *you/does [name of Patient]]* have? | • Medicare Advantage<br>• Medicare supplement<br>• Medicaid<br>• Military related health care including TRICARE, CHAMPUS, VA health care and CHAMP-VA<br>• Indian Health Service<br>• A state-sponsored health plan<br>• Other government program<br>• Other [insert open text box]<br>• No insurance<br>• Prefer not to say | |
| Confirmation of SNF/HHA Care | Can I confirm that *[you/ [name of Patient]]* received care from [name of SNF/HHA] after a recent hospitalization? | • *[insert text]* | P, F |
| Length of SNF/HHA Care | About how long *[were [you/they] at [name of SNF]/ did [you/they] receive home care from [name of HHA]]*? | • *[insert number]* | P, F |
| Prior Use of SNF/HHA Services | Have *[you/they]* been admitted to a rehabilitation facility like [name of SNF] before?/ Was this *[your/their*] first time using home health services? | • Yes<br>• No | P, F |
| **Employment** | | | |
| Job role | What is your position or official job title at your organization? | • *[insert text]* | A |
| Job role | Can I confirm that you are the/a _____ [position in the facility/organization]? | • *[insert text]* | S |
| Time at organization | How long have you worked at your organization, in years and months? | • *[insert number]* | A |
| Time at organization | How long have you been working for [name of organization]? | • *[insert number]* | S |
| Prior position | If employment with organization is less than 2 years, did you hold a similar position at another organization? | • Yes<br>• No | A, S |
| Time in Healthcare | How long have you worked in healthcare? | • *[insert number]* | S |

| | | | |
|---|---|---|---|
| Hours worked | How many hours a week do you work? | • *[insert number]* | A, S |
| Employment status | [In response to hours worked a week] is this considered full time or something else? | • Full time<br>• Part-time<br>• Casual<br>• Other | A, S |

Note: Interview type includes A= Administrator, S= frontline staff of the HHA or SNF, P= SNF or HHA patient, F= family caregiver of the SNF or HHA patient
