# Supplementary material for: Implementation of US Post-Acute Care Payment Reform and COVID-19 Policies: Examining Experiences of Health System Leaders, Staff, Patients, and Family Caregivers—A Protocol
Source: Int J Environ Res Public Health. 2023 Oct 23;20(20):6959. doi: 10.3390/ijerph20206959 (PMC10606322; doi:10.3390/ijerph20206959)
Supplement: Supplementary file 1 [file ijerph-20-06959-s001.zip › Supplemental File S6 final NEL.pdf]

Supplemental File S6: SNF and HHA Organizational Characteristics

Domain	Data Source	Description of variables
**NH Characteristics**		
Region	CMS Provider of Services File	• Northeast, South, Midwest, West
State	CMS Provider of Services File	• 2 letter state code, indicating the state in the US where the SNF/HHA is located
Urbanicity indicator based on Core Based Statistical Area codes	CMS Provider of Services File	• Urban<br>• Rural
Profit status	CMS Provider of Services File	• For profit<br>• Non-profit<br>• Government owned
Chain affiliation	Long Term Care Focus	• Dichotomous indicator for SNF being part of a chain
Branch affiliation	CMS Provider of Services File	• Indicator of HHAs that operates branches (not the same as chain status)
SNF within a Hospital	CMS Care Compare for SNF	• Dichotomous indicator for a SNF that is located within a hospital
Size	CMS Provider of Services File	• Number of certified beds for SNFs<br>• Number of episodes for HHAs
CMS quality 5-star rating for HHA	CMS Care Compare for HHA	• 1-5 stars
CMS overall 5-Star rating for SNF	CMS Care Compare for SNF	• 1-5 stars
CMS staffing 5-star rating for SNF	CMS Care Compare for SNF	• 1-5 stars
CMS short-stay quality measures 5-star rating for SNF	CMS Care Compare for SNF	• 1-5 stars
CMS health inspections 5-star rating for SNF	CMS Care Compare for SNF	• 1-5 stars
Certification of need (CON)	National Conference of State Legislatures	• Dichotomous indicator for whether SNF/HHA is located in a state with a CON law
Moratorium in place	National Conference of State Legislatures	• Dichotomous indicator for whether SNF is located in a state with a moratoria in place
CMS certification	CMS Provider of Services File	• Years certified as a SNF or HHA based on initial date of CMS certification

Staffing levels for SNF	Payroll Based Journal	• Nursing hours per resident per day<br>• Rehabilitation staff hours per resident per day
Use of contract (or in-house) therapy for SNF	Payroll Based Journal	• All contract therapy staff<br>• All in-house therapy staff<br>• Mix
County-level COVID indicators	USAFacts	• County-level rates of COVID cases<br>• County-level rates of COVID deaths
Aggregate patient-level measures		
% of patients dually eligible for Medicare & Medicaid	Medicare Post-Acute Care and Hospice Provider Utilization and Payment Public Use Files	• Percent of Medicare beneficiaries (short-stay residents for SNFs, all patients for HHAs) annually who are dually eligible for Medicaid
% of patients that reside in rural underserved regions	Medicare Post-Acute Care and Hospice Provider Utilization and Payment Public Use Files	• Percent of Medicare beneficiaries (short-stay residents for SNFs, all patients for HHAs) annually who receive their Medicare correspondence in a ZIP designated as rural
% Asian or Pacific Islander	Medicare Post-Acute Care and Hospice Provider Utilization and Payment Public Use Files	• Percent of Medicare beneficiaries (short-stay residents for SNFs, all patients for HHAs) annually who are Asian or Pacific Islanders
% American Indian or Alaska Native	Medicare Post-Acute Care and Hospice Provider Utilization and Payment Public Use Files	• Percent of Medicare beneficiaries (short-stay residents for SNFs, all patients for HHAs) annually who are American Indian or Alaska Native
% non-Hispanic Black patients	Medicare Post-Acute Care and Hospice Provider Utilization and Payment Public Use Files	• Percent of Medicare beneficiaries (short-stay residents for SNFs, all patients for HHAs) annually who are non-Hispanic Black or African American
% Hispanic patients	Medicare Post-Acute Care and Hospice Provider Utilization and Payment Public Use Files	• Percent of Medicare beneficiaries (short-stay residents for SNFs, all patients for HHAs) annually who are Hispanic

% Other race	Medicare Post-Acute Care and Hospice Provider Utilization and Payment Public Use Files	• percent of Medicare beneficiaries (short-stay residents for SNFs, all patients for HHAs) annually with race not elsewhere classified or unknown
% White patients	Medicare Post-Acute Care and Hospice Provider Utilization and Payment Public Use Files	• percent of Medicare beneficiaries (short-stay residents for SNFs, all patients for HHAs) annually who are non-Hispanic white
